# A new visual index for assessing zooplankton biomass and its utility in assessing prey availability for megaplanktivores

**DOI:** 10.1093/plankt/fbag027

**Published:** 2026-05-27

**Authors:** Hannah M Moloney, Asia O Armstrong, Guy M W Stevens, Christine L Dudgeon, Joanna L Harris, Kathy A Townsend, Anthony J Richardson

**Affiliations:** School of Science, Technology and Engineering, University of the Sunshine Coast, Fraser Coast Campus, 161-179 Old Maryborough Road Hervey Bay, Queensland 4655, Australia; The Manta Trust, Catemwood House, Norwood Lane, Corscombe, Dorset DT2 0NT, United Kingdom; Maldives Manta Conservation Programme, M. Kureli, Buruzu Magu, Maafannu, 20304 Malé, Kaafu Atoll, Republic of Maldives; Commonwealth Scientific and Industrial Research Organisation (CSIRO) Environment, BioSciences Precinct (QBP), 306 Carmody Road St Lucia, Queensland 4067, Australia; School of Science, Technology and Engineering, University of the Sunshine Coast, Moreton Bay Campus, 1 Moreton Parade Petrie, Queensland 4502, Australia; IUCN SSC Shark Specialist Group, P.O. Box 29588, Dubai, United Arab Emirates; The Manta Trust, Catemwood House, Norwood Lane, Corscombe, Dorset DT2 0NT, United Kingdom; Maldives Manta Conservation Programme, M. Kureli, Buruzu Magu, Maafannu, 20304 Malé, Kaafu Atoll, Republic of Maldives; School of Science, Technology and Engineering, University of the Sunshine Coast, Moreton Bay Campus, 1 Moreton Parade Petrie, Queensland 4502, Australia; Biopixel Oceans Foundation, 14-16 Church Street Fortitude Valley, Queensland 4006, Australia; The Manta Trust, Catemwood House, Norwood Lane, Corscombe, Dorset DT2 0NT, United Kingdom; School of Biological and Marine Sciences, University of Plymouth, Drake Circus, Plymouth, Devon, PL4 8AA, United Kingdom; School of Science, Technology and Engineering, University of the Sunshine Coast, Fraser Coast Campus, 161-179 Old Maryborough Road Hervey Bay, Queensland 4655, Australia; Commonwealth Scientific and Industrial Research Organisation (CSIRO) Environment, BioSciences Precinct (QBP), 306 Carmody Road St Lucia, Queensland 4067, Australia; School of the Environment, The University of Queensland, St Lucia, Queensland 4072, Australia

**Keywords:** plankton, blooms, marine, planktivores, prey abundance

## Abstract

Zooplankton are vital links in marine food webs, yet their biomass remains challenging to quantify across broad spatial and temporal scales. This study introduces the Zooplankton Visual Index–a simple, rapid and scalable semi-quantitative method for evaluating zooplankton biomass through underwater observations. Following the establishment of a standardized protocol, validation against *in situ* net samples demonstrated that index levels increased consistently with measured biomass and predictable shifts in community composition. We then applied the validated index to a 9-year dataset of reef manta ray (*Mobula alfredi*) sightings in the Maldives. Increased *M. alfredi* abundance significantly correlated with higher index levels, confirming that the index effectively captures the food environment as an important predictor of behavior and habitat use. Thus, the Zooplankton Visual Index provides an accessible and validated tool for assessing prey availability, enabling researchers, non-specialist field staff and citizen science programs to contribute to broad-scale ecological research and conservation efforts for marine megaplanktivores.

## INTRODUCTION

Zooplankton are key consumers in marine ecosystems, linking primary productivity to higher trophic levels (Wimalasiri *et al.,* 2021) and ensuring the continued functioning of ocean food webs (Richardson, 2008). They are a vital food source for a diverse array of predators, including fishes, sea turtles, marine mammals, seabirds (Frederiksen *et al.,* 2006), and megaplanktivores such as baleen whales, basking sharks, whale sharks and mobulid rays (Sims, 1999; Goldbogen *et al.,* 2006; Nelson and Eckert, 2007; Rohner *et al.,* 2017). Despite their critical ecological role, many studies do not estimate zooplankton abundance directly, instead using phytoplankton biomass from satellite-derived chlorophyll-*a* (Chl-*a*) concentrations as a proxy (Anderson *et al.,* 2011; Lambert *et al.,* 2014; Harris *et al.,* 2020; Ahsin *et al.,* 2022; Cabral *et al.,* 2023). Consequently, many movement and foraging studies often assume zooplankton biomass is directly related to Chl-*a* concentrations (e.g. Anderson *et al.,* 2011; Harris *et al.,* 2020; Ahsin *et al.,* 2022). However, Chl-*a* is often a poor proxy for zooplankton biomass (e.g. Wei *et al.,* 2022; Renaud *et al.,* 2024). While correlated at broad time and space scales (Richardson and Schoeman, 2004), the relationship breaks down at finer scales due to their differing growth rates, turnover times, mobility and the varying effects of bottom-up and top-down control of food webs (Lalli and Parsons, 1997). Thus, direct measurements are more robust than relying on satellite-derived proxies.

Estimating zooplankton biomass is challenging; it is rarely detectable by satellite (except in rare cases such as dense *Calanus* or krill swarms, Basedow *et al.,* 2019) and *in situ* collection can be logistically difficult. The traditional *in situ* method for estimating zooplankton biomass involves net sampling, typically through boat-based tows (Harris *et al.,* 2000; Rohner *et al.,* 2015; Armstrong *et al.,* 2016; D’Agostino *et al.,* 2024) followed by weighing in the laboratory (Postel *et al.,* 2007). Modern instruments, such as Optical Plankton Counters, Video Plankton Recorders, Underwater Vision Profilers, Acoustic Doppler Current Profilers and multi-frequency echosounders, can estimate zooplankton biomass (Remsen *et al.,* 2004; Davis *et al.,* 2005; Dunn and Zedel, 2022; Picheral *et al.,* 2022) but are often imprecise, costly and require sophisticated tools such as artificial intelligence for converting data into biomass (Lombard *et al.,* 2019; D’Antonio *et al.,* 2024). Because such methods are not always feasible or affordable for monitoring programs, a simple, rapid and cost-effective alternative for evaluating zooplankton biomass would be valuable.

Visual approaches might offer an alternative, albeit at a coarser scale. For estimating phytoplankton biomass, examples include Secchi depth for water transparency and Chl-*a* estimation (Boyce *et al.,* 2010), the Forel-Ule scale for water color assessment (Ye and Sun, 2022), the phytoplankton color index for surface Chl-*a* derived from continuous plankton recorder samples (Richardson *et al.,* 2006), and the visual cyanobacteria index for classifying cyanobacterial blooms (Oyama *et al.,* 2015). Several of these visual methods have been instrumental in monitoring long-term environmental trends in phytoplankton, assessing water quality and facilitating broad-scale data collection, often through citizen science initiatives (Reid *et al.,* 1998; Richardson *et al.,* 2006; Oyama *et al.,* 2015; Ye and Sun, 2022). In contrast to phytoplankton, visual indices for zooplankton remain rare.

The few visual indices that have been described and used to predict megaplanktivore abundance and behavior, generally remain unvalidated against physical zooplankton biomass samples (Rohner *et al.,* 2013; Stevens, 2016; Hildebrand *et al.,* 2022; D’Agostino *et al.,* 2024; Murray *et al.,* 2024; Venables *et al.,* 2024; Dawn *et al.,* 2025). These indices generally fall into two categories: in-water observer assessments and camera/video-based estimates. In-water observer indices typically use three to four ordinal levels, and have linked higher estimated zooplankton densities to increased number of foraging reef manta rays (*Mobula alfredi*) in the Maldives (Murray *et al.,* 2024) and Mozambique (Rohner *et al.,* 2013). Interestingly, one study in Mozambique found that oceanic manta rays (*Mobula birostris*) frequented cleaning stations rather than undertook feeding when zooplankton concentration was low, suggesting behavioral shifts under sub-optimal conditions (Venables *et al.,* 2024).

Camera-based indices, often relying on video stills or attached tags (e.g. The Crittercam from National Geographic), have identified high-density zooplankton patches utilized by gray whales (*Eschrichtius robustus*) (Hildebrand *et al.,* 2022; Dawn *et al.,* 2025), right whales (*Eubalaena australis*) (D’Agostino *et al.,* 2024) and *M. alfredi* (Stewart *et al.,* 2019). Crucially, despite their utility, these visual indices are limited by a lack of validation against concurrent physical zooplankton samples and a lack of standardized, repeatable protocols.

Here, we introduce the Zooplankton Visual Index (ZVI), a simple, rapid, scalable and semi-quantitative method for estimating *in situ* zooplankton biomass. We present the ZVI protocol and validate it against zooplankton biomass measured from concurrently collected net samples. We then apply the validated ZVI to a 9-year dataset of manta ray sightings to assess whether higher zooplankton biomass estimated from the ZVI corresponds to increased manta ray abundance. We hope that the ZVI has utility in both researcher-led and citizen science initiatives, particularly in nations with limited resources.

## METHODS

### Study location

Situated 422 km southwest of India, the Republic of Maldives is a small island nation in the central Indian Ocean with 26 geographical atolls extending 870 km north to south (Harris *et al.,* 2020). Data in the present study was collected in the Hanifaru Marine Protected Area (MPA; 5.1733°N, 73.145°E), Baa Atoll. This site consistently concentrates dense patches of zooplankton during the Southwest Monsoon (*Hulhaangu*; May–November), which in turn attracts megaplanktivores including *M. alfredi* (Stevens, 2016; Harris *et al.,* 2020; Armstrong *et al.,* 2021; The Manta Trust, 2024) and whale sharks (*Rhincodon typus;*  Neves, 2009). This site was chosen because the zooplankton density regularly fluctuates between very high and low values, and there is a long-standing research program with a 9-year dataset involving citizen scientists to document and understand mass aggregations of foraging megaplanktivores, especially *M. alfredi*.

### Study design

#### The zooplankton visual index protocol

**Fig. 1:**
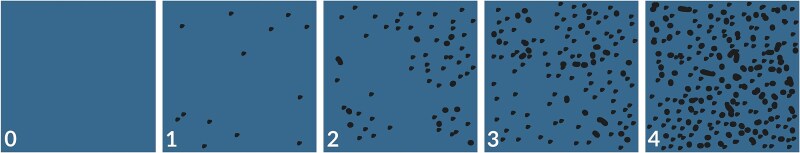
Standardized density charts used to assess zooplankton visually in the water. The Zooplankton Visual Index levels on the charts are: (0) apparent absence of zooplankton, (1) thin layer or small patch of zooplankton, (2) multiple layers or patches of zooplankton, (3) water is thick and cloudy, with zooplankton felt on skin, (4) water is dense and “soup-like.”

The ZVI was developed to provide a standardized method to estimate zooplankton density underwater. Observers, in the water on snorkel, assess the ZVI by spending ~ 30 s slowly rotating in place, visually inspecting the water column in all directions within ~ 2 m radius. Zooplankton abundance is then estimated based on the reference zooplankton density charts (Fig. 1). The index has five levels of zooplankton density: (0) apparent absence of zooplankton; (1) a thin layer or small patch of zooplankton; (2) multiple layers or patches of zooplankton; (3) water appears thick and cloudy, with zooplankton felt on the skin; (4) water is dense and “soup-like” (Fig. 1).

ZVI assessments were conducted during daylight hours and only when weather conditions were safe for observers to do so (i.e. < 7 on the Beaufort scale). Due to the natural patchiness of zooplankton, large size of the site (439 823 m^2^), and the high temporal variation of zooplankton density over a 12-h period (Armstrong *et al.,* 2021), the overall mean zooplankton density was obtained per survey by all in-water observers (1–7 observers) present during that survey period. This meant that for each survey there was a single ZVI value.

All observers undergo training with experienced practitioners to ensure consistency and accuracy in ZVI estimates and to reduce subjectivity across observers (Standard Operating Procedure; Supplementary 1). Training consisted of both verbal and written instructions. In addition, for researchers there was in-water training, followed by an in-water assessment. New observers were required to make three ZVI assessments that matched the trainers’ assessments before their observations were considered. However, training for citizen scientists consisted of only verbal and written instructions because the in-water training facet was not feasible.

#### Validation of the zooplankton visual index

To validate ZVI estimates, concurrent *in situ* net samples of zooplankton were collected between May–November in 2017 (*n* = 78), 2021 (*n* = 49), 2022 (*n* = 59), and 2023 (*n* = 65). Samples were collected using a 200-μm mesh net with a 30-cm diameter mouth and fitted with a flowmeter to allow calculation of the volume of water sampled. The net was hand-towed horizontally for a mean of 3.3 min ±0.20 *SE* against the current at a speed of ~ 2 knots by snorkelers; this was necessary because boat use is restricted in Hanifaru MPA due to MPA regulations (see Armstrong *et al.,* 2021). Prior to use in the field, the flowmeter was calibrated in a swimming pool of known length to establish accurate measurement of distance per flowmeter revolution. The *in situ* net samples and ZVI estimates, collected during sampling surveys, were collected from a range of matching depths (0.5–18 m). The net was towed for a minimum of ~ 20 m and filtered between ~ 1.5 and 15 m^3^ of seawater. The volume of water filtered varied with the depth of sample collection (i.e. it is physically more feasible to conduct a longer tow at the surface compared to freediving at depth), selected tow route (i.e. dependent spatially on where *M. alfredi* individuals were), and other obstacles such as tourist snorkelers and animals. The abundance of *M. alfredi,* estimated as the number of individuals counted, were also recorded during these sampling surveys to test the application of the ZVI method.

Contents of the net samples were kept on ice, rinsed with freshwater and either fixed with 10% buffered formalin solution or oven dried at 60°C for 24 h at the end of each day at a laboratory facility in the Maldives. Samples preserved in formalin were later oven dried at 60°C for 24 h to measure zooplankton dry weight (DW) (Armstrong *et al.,* 2021) at a laboratory facility in Australia. The biomass per unit volume of water filtered (mg m^−3^) for each individual net tow was calculated by dividing the DW of the sample (mg) by the volume of water filtered (m^3^).

#### Application of the zooplankton visual index to long-term manta ray monitoring

Long-term logbook data is a common method used to investigate trends in sightings, movement and behavior of megafauna (Watson *et al.,* 2001; Jaine *et al.,* 2012; Rohner *et al.,* 2013; Armstrong *et al.,* 2016; Pirotta *et al.,* 2020; Venables *et al.,* 2024). Long-term data were recorded on *M. alfredi* sightings and population dynamics, ZVI and prey community composition (dataset hereafter referred to as logbook data; The Manta Trust, 2024). Logbook data was curated by the *Maldives Manta Conservation Program* and *The Manta Trust,* and was collected by trained researchers and citizen scientists (https://www.mantatrust.org/maldives; The Manta Trust, 2024). The logbook data comprised 1261 surveys at Hanifaru MPA spanning 9 years (May 2016–November 2024). Sightings of *M. alfredi* were estimated as the number of individuals per survey. The prey abundance was recorded as ZVI level (0–4) during surveys. To assess whether there were potential differences between researchers and citizen scientists in their ZVI scores, data was categorized as either from researcher surveys (*n* = 1176; 2016–2024) or citizen scientist surveys (*n* = 85; 2019–2023). Both datasets are non-overlapping, with both collecting the ZVI and *M. alfredi* abundance over the duration of the survey (mean survey duration: researcher surveys = 123.2 min ±2.44 *SE*, range 8–300 min; citizen scientist surveys = 45.4 min ±2.37 *SE*, range 5–215 min). Citizen scientists were most commonly industry professionals (i.e. dive instructors, guides, resort marine biologists) who worked for tour operator companies that offered snorkeling and diving tours to their guests.

The zooplankton community composition from researcher surveys (*n* = 1172) was used to investigate the relative importance of groups of zooplankton as prey for *M. alfredi*. For each survey, trained observers reported the broad zooplankton taxonomic groups (i.e. chaetognaths, copepods, ctenophores, fish larvae, isopods, jellyfishes, ostracods, salps and shrimp-like organisms) present. This identification cannot be validated as there were no physical samples collected concurrently with the composition assessments. Zooplankton groups that had ≤ 40 observations were excluded from data visualization (e.g. isopods).

In addition to the logbook data, sampling surveys were conducted specifically during the collection of physical zooplankton samples for ZVI validation. While these were also performed by trained researchers, they differed fundamentally from standard researcher surveys in their temporal scale. Each sampling survey was a high-intensity observation nested within a broader researcher survey, occurring only during the specific timeframe of the net tow (mean survey duration: 3.3 min ±0.20 *SE*, range: 0.5–27 min). Abundance of *M. alfredi* during the sampling survey was estimated by counting the unique individuals sighted within the immediate vicinity of the net (generally within a < 25 m radius). To account for the discrepancy in effort between these short-duration sampling surveys and the standard researcher surveys, a Welch’s *t-*test was used to compare sighting rates (number *M. alfredi* per minute) between the two researcher-led methods (i.e. *P* < 0.05).

### Statistical analysis

A Generalized Linear Model (GLM) using the statistical software R version 4.4.2 (R Core Team 2024) was used to validate the ZVI method. The GLM had zooplankton biomass (mg m^−3^) as the response and three fixed effect predictors. These were ZVI (with five levels), Location (where the zooplankton biomass samples were measured, with two levels representing the Maldives or Australia), and the Location:ZVI interaction. Because the response is continuous, positive, and non-zero and the residuals increased with the mean, we used a Gamma error distribution with a log link function.

To assess whether the number of *M. alfredi* in Hanifaru MPA was related to zooplankton biomass as estimated by the ZVI, we analyzed logbook data from observer trips from 2016-2024. There was usually one (occasionally two) trip per day, 6 days a week, during the manta ray season (May-November). We constructed a GLM with estimated *M. alfredi* number as the response, with two main effects and their interaction. These were ZVI (with five levels), Survey Type (with three levels representing sampling survey, researcher survey and citizen science survey) and their interaction. Because the response is a count, we first tried a Poisson error structure but it was overdispersed, so we then used a negative binomial error structure (in the MASS R package; Venables and Ripley, 2022) with a log link function, which was not overdispersed.

For both GLMs, model residuals were visually assessed for normality and homogeneity of variance. Significance of interactions and main effects was assessed using an analysis of deviance based on a likelihood ratio test.

## RESULTS

### Validation of the zooplankton visual index

**Fig. 2:**
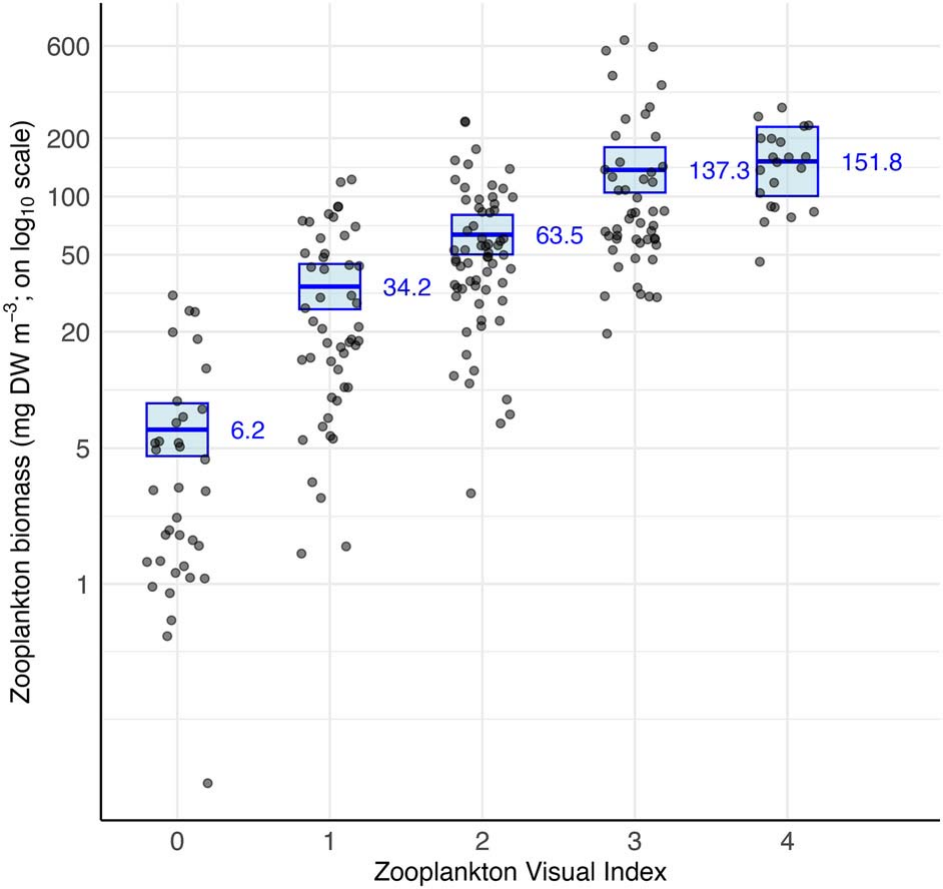
Predicted zooplankton biomass (mg DW m^−3^; mean ± 95% confidence intervals) against the reported Zooplankton Visual Index (ZVI) from the final GLM using a log link function. Raw data are represented by black dots (*n* = 251).

Zooplankton biomass was positively and significantly related to ZVI (F_4, 214_ = 47.465, *P* < 0.001) (Fig. 2). Mean estimated zooplankton biomass increased across ZVI levels: level 0 (6.2 mg DW m^−3^), level 1 (34.2 mg DW m^−3^), level 2 (63.5 mg DW m^−3^), level 3 (137.3 mg DW m^−3^) and level 4 (151.8 mg DW m^−3^). There was no significant interaction between the location of the laboratory and zooplankton biomass (F_4, 209_ = 2.3196, *P* = 0.0581), thus the interaction was dropped from the final model, leaving main effects only.

### Are long-term manta ray sightings related to the zooplankton visual index?

**Fig. 3:**
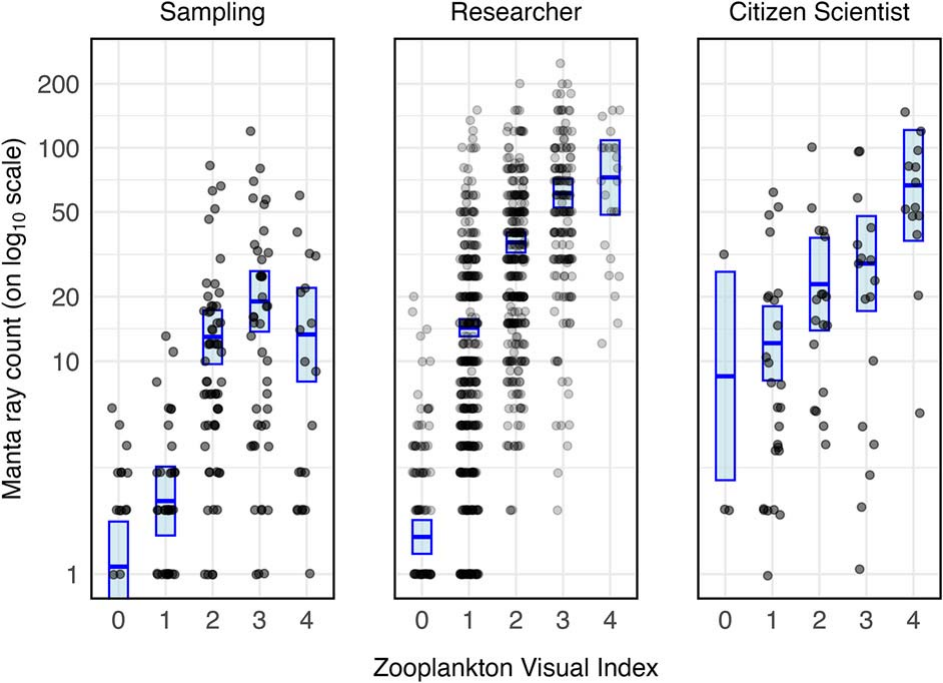
Predicted reef manta ray *(Mobula alfredi)* abundance (presented on the log-scale; mean ± 95% confidence intervals shaded) against the reported Zooplankton Visual Index (ZVI) from the final GLM using a log link function and interaction between survey type and Zooplankton Visual Index (ZVI). The raw data points are shown in black for researcher surveys (2016–2024, *n* = 1176), citizen scientist surveys (2019–2023, *n* = 85) and sampling surveys (2017, 2021–2023, *n* = 251). All three categories on separate panels show a positive trend from levels 0 to 3. The sampling survey line is significantly steeper than the others but shows a slight decrease in abundance at level 4, while the researcher and citizen scientist lines continue to trend upward.

*Mobula alfredi* abundance was positively and significantly related to ZVI level, while accounting for the effect of the various survey types (sampling surveys, researcher surveys, citizen science surveys; Fig. 3). There was a significant interaction between the type of survey and ZVI estimates (χ^2^(8) = 51.01, *P* < 0.001), indicating that the effect of zooplankton on *M. alfredi* abundance differed significantly among the three observer groups of survey types, despite both sampling and researcher surveys being conducted by researchers. There was a significant difference in *M. alfredi* sighting rates between the two researcher-led survey methods (Welch’s *t-*test: *t*_218.1_ = −6.49, *P* < 0.001), justifying these categories being treated separately in the analysis. Although researcher surveys recorded higher raw counts of *M. alfredi* over their longer durations (mean = 0.21 ± 0.009 *SE* sightings per minute), the sighting rate was significantly higher in the targeted sampling surveys (mean = 3.89 ± 0.568 *SE* sightings per minute). This suggested that while all groups observed a positive trend between ZVI and *M. alfredi* sightings, the magnitude of the effect was influenced by survey design and effort.

There was a general trend of increasing *M. alfredi* abundance with higher ZVI levels consistent across researcher and citizen scientist surveys, with the exception of sampling surveys, where *M. alfredi* abundance decreased slightly from ZVI level 3 to 4 (Fig. 3). Specifically, the mean estimated *M. alfredi* abundance in researcher surveys for high levels of zooplankton (ZVI level 4) were 4.8 times higher than for lower levels of zooplankton (ZVI level 1). These same surveys also had a 10-fold increase in the mean estimated *M. alfredi* abundance when there was a thin layer of zooplankton present (mean ZVI 1 = 15.2 ± 0.7 *SE*) compared to surveys that had no apparent visible zooplankton (mean ZVI 0 = 1.5 ± 0.2 *SE*).

### Major prey items

**Fig. 4:**
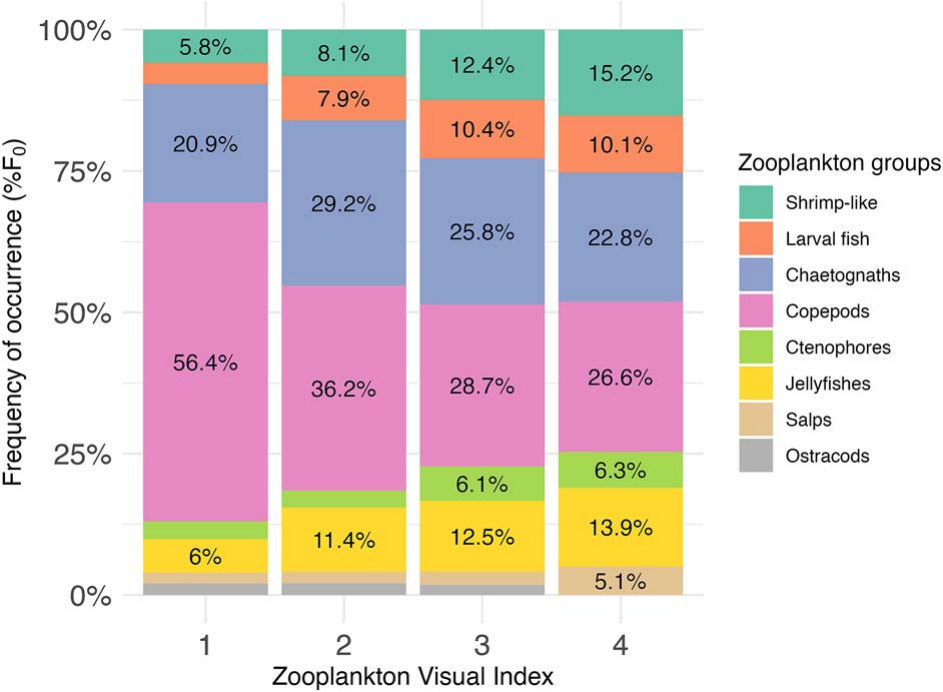
Zooplankton composition (%) based on presence or absence of zooplankton groups in relation to the Zooplankton Visual Index during researcher surveys (*n* = 1172). Data displayed as the percentage frequency of occurrence (%F_0_).

During researcher surveys, all zooplankton groups were represented at all ZVI levels (except for ostracods in ZVI level 4; Fig. 4). However, the community composition overall was dominated by copepods, with 75.3% of recorded estimates containing the taxa (*n* = 883). The frequency of occurrence of copepods in samples declined as ZVI levels increased, due to other taxa being observed more frequently and the composition becoming more evenly distributed (Fig. 4).

## DISCUSSION

Current methods for estimating zooplankton biomass are often cost-prohibitive and logistically demanding (Postel *et al.,* 2007). The validation of the ZVI against *in situ* biomass and the shifting community composition establishes the index as a robust, semi-quantitative alternative to traditional net sampling. By bridging the gap between visual observations and physical samples, the ZVI provides a feasible proxy for assessing prey availability in field surveys where laboratory processing or specialized equipment is unavailable. This tool enables the collection of extensive, standardized datasets across spatial and temporal scales previously inaccessible to traditional methods.

The application of the ZVI to *M. alfredi* monitoring at Hanifaru MPA demonstrates its utility in characterizing the functional link between the food environment and predator ecology. The strong correlation between ZVI levels and *M. alfredi* sightings confirms that the index effectively captures the environmental drivers of predator behavior and habitat use. The marked 10-fold increase in *M. alfredi* abundance at even the lowest ZVI level (level 1) from an apparent absence of zooplankton (level 0), suggests a high sensitivity to subtle changes in food availability, identifying a potential foraging threshold for this threatened species. Further, the consistency of this trend across observer groups–including researchers and citizen scientists–underscores the ZVI’s reliability for broad-scale monitoring and conservation management.

### Prey density and composition for manta rays

Planktivores require a minimum prey density to meet energetic requirements that trigger foraging. This has been documented in fin whales (*Balaenoptera physalus*) (Goldbogen *et al.,* 2007)*,* whale sharks (*R. typus*) (Nelson and Eckert, 2007), and basking sharks (*Cetorhinus maximus*) (Sims, 1999). In the current study, the mean biomass for ZVI level 1 (34.2 mg DW m^−3^) aligns closely with previously estimated theoretical foraging thresholds for *M. alfredi* on the Great Barrier Reef in Australia (25.2 mg DW m^−3^; Armstrong *et al.,* 2016), and in the Seychelles (26.9 mg DW m^−3^; Pouponeau *et al.,* 2026). Notably, the shift from a level 0 to level 1 drove the largest proportional increase in *M. alfredi* abundance across our dataset. As biomass nearly doubled again at ZVI level 2, *M. alfredi* abundance similarly more than doubled (from 15 to 37 individuals). This level 2 mean (63.5 mg DW m^−3^) aligns with the previously calculated prey density threshold specifically for Hanifaru MPA (53.7 mg m^−3^; Armstrong *et al.,* 2021). This helps explain the large aggregations of *M. alfredi* observed at Hanifaru MPA during the Southwest Monsoon, when nutrient upwelling drives the increased Chl-*a* concentrations associated with the down-current side of the atoll (Harris *et al.,* 2020).

The transition from copepod dominance at lower ZVI levels to a more taxonomically diverse, evenly distributed community at higher ZVI levels when dense swarms were observed, suggests the ZVI effectively captures shifts in community structure as biomass increases. This broad taxonomic representation aligns with previous studies in Hanifaru MPA, where copepods typically dominate the foraging environment (Armstrong *et al.,* 2021), as well as studies of *M. birostris* in Ecuador (Burgess, 2017) and *C. maximus* in the UK (Sims, 1999). While the specific dominant taxa in the food environment for *R. typus* varies–ranging from larval fishes and bait fishes in Honduras (Fox *et al.,* 2013), to shrimp-like organisms (sergestid shrimp) in Tanzania (Rohner *et al.,* 2015), and a mix of sergestid shrimp, copepods, chaetognaths and larval fish in Mexico (Motta *et al.,* 2010) – the underlying driver for megaplanktivore foraging appears to be the prey density rather than taxonomic selectivity. Consequently, the ZVI serves as a robust proxy for the density thresholds required to trigger feeding behavior, identifying “energy hotspots” regardless of the specific prey groups involved.

### Benefits of using the zooplankton visual index

While less precise than physical sampling, the ZVI offers several distinct advantages. First, it enables the cost-effective collection of extensive datasets across spatial and temporal scales that are often logistically and financially prohibitive for traditional methods. Second, whereas measured biomass requires specialized equipment and laboratory processing (Postel *et al.,* 2007), the ZVI requires minimal training. This accessibility allows for data collection alongside other research activities by a broad range of stakeholders, including government teams, tourism operators and citizen scientists–engagements that also promote environmental stewardship (Earp and Liconti, 2020). Finally, traditional net samples can be unrepresentative due to zooplankton patchiness (Richardson *et al.,* 2006). By integrating observations across the entire water column (in shallow areas) and survey duration, the ZVI provides a mean index that may better account for the spatial and temporal heterogeneity of prey. Ultimately, this validated visual index enhances megafauna behavioral studies and provides a scalable framework for monitoring prey in regions where traditional sampling is logistically or financially unfeasible.

### Caveats of using the zooplankton visual index

There are several important caveats to consider when using the ZVI to estimate zooplankton biomass. First, the ZVI provides a coarse, rapid estimate of zooplankton density. While it offers practical advantages in terms of speed and ease of use, this comes at the cost of reduced precision compared to laboratory-processed samples. Nonetheless, the strong positive correlation between the ZVI and concurrently measured zooplankton biomass – as well as the observed relationship between ZVI levels and the number of *M. alfredi* sightings – supports its utility as a reliable proxy. Second, water clarity influenced by phytoplankton or suspended sediment is a constraint for any visual assessment method. In the current study, this impact was likely minimal; the Maldives is an oligotrophic reef system (Su *et al.,* 2021), and water visibility was < 5 m in only 2.4% of the 991 researcher surveys where visibility was recorded. Further, the high variability of ZVI levels (0–3) recorded during these rare low-visibility events suggests that biomass detection was not visibility-limited at this site. However, for broader application in more turbid or productive environments – such as upwelling zones or estuaries – the ZVI framework may require site-specific calibration. In such regions, high concentrations of suspended sediment or marine snow could lead to biomass overestimation if abiotic particles are misidentified as zooplankton. Conversely, reduced detection volumes in low-visibility water might lead to underestimation. In these contexts, we recommend that the ZVI be initially validated against concurrent net samples to adjust for local optical conditions. Because the ZVI is based on the density of zooplankton patches immediately surrounding the observer, rather than how far an observer can see through the water, it remains a promising tool for use in various environments.

Third, both the community composition and vertical distribution of zooplankton in the water column could influence ZVI estimates. Larger or more opaque zooplankton (e.g. copepods) could visually elevate ZVI levels, whereas smaller (<1 mm) or more translucent taxa (e.g. chaetognaths) may result in lower perceived ZVI levels. Interestingly, copepods–despite their opacity–declined in relative abundance with increasing ZVI levels, compared to the more translucent taxa. Last, site-specific physical characteristics, such as the bathymetry, benthic habitat type and depth, may also impact ZVI estimates by either enhancing or obscuring visual detection of zooplankton. This is unlikely to be a confounding factor in the present study, as Hanifaru MPA features a shallow (<20 m), predominantly sandy, seabed that facilitates visual assessment. However, when applying the ZVI in other locations, these factors should be carefully considered as their influence may vary depending on local environmental conditions.

## CONCLUSION

This study validates the ZVI protocol as a rapid, scalable and semi-quantitative alternative to traditional net sampling for estimating zooplankton biomass in surface waters. Consistent increases in measured biomass and predictable shifts in community composition across ZVI levels confirm the index as a robust ecological proxy. Consequently, the ZVI provides a practical tool for monitoring food availability and habitat use for megaplanktivores. Given that higher ZVI levels directly correspond to increased *M. alfredi* abundance, this protocol offers an accessible framework for both researchers and citizen science initiatives to contribute to broad-scale ecological research and conservation efforts.

## Data Archiving

The datasets generated and analyzed during the current study – zooplankton sample data (including biomass and ZVI observations) and logbook data (including manta ray abundance, zooplankton taxonomy and ZVI observations) – are available on reasonable request from the corresponding author.

## Supplementary Material

Moloney_et_al_Zooplankton_visual_index_Supplementary_fbag027
