# Supplementary material for: A new visual index for assessing zooplankton biomass and its utility in assessing prey availability for megaplanktivores
Source: J Plankton Res. 2026 May 27;48(3):fbag027. doi: 10.1093/plankt/fbag027 (PMC13215585; doi:10.1093/plankt/fbag027)
Supplement: Moloney_et_al_Zooplankton_visual_index_Supplementary_fbag027 [file moloney_et_al_zooplankton_visual_index_supplementary_fbag027.docx]

Supplementary

**SUP 1. Standard Operating Procedures for Zooplankton Visual Index data collection**

*11 August 2025*

Table of Contents

[1. SCOPE AND APPLICATION 1](#_Toc212446440)

[2. SUMMARY OF METHOD 1](#_Toc212446441)

[3. SAFETY 2](#_Toc212446442)

[4. EQUIPMENT & DATA RECORDING REQUIREMENTS 2](#_Toc212446443)

[5. TRAINING 2](#_Toc212446444)

## 1. SCOPE AND APPLICATION

1.1 This Standard Operating Procedure describes the field sampling techniques to collect Zooplankton Visual Index (ZVI) data in shallow-water marine ecosystems. The ZVI estimates zooplankton density underwater and can be used to assess the relative abundance of prey for planktivorous animals.

## 2. SUMMARY OF METHOD

2.1 Select a site that is of interest for your research objectives and is regularly visited.

2.2 Observers should enter the water and wait 3 minutes for water disturbances to settle in

preparation to assess the ZVI.

2.3 Observers are to assess the ZVI by spending ~30 seconds slowly rotating in place, visually inspecting the water column in all directions. Zooplankton abundance is then compared against reference zooplankton density charts (Fig. 1). The index has five levels: (0) apparent absence of zooplankton; (1) a thin layer or small patch of zooplankton; (2) multiple layers or patches of zooplankton; (3) water appears thick and cloudy, with zooplankton felt on the skin; (4) water is dense and ‘soup-like’. Due to the natural patchiness of zooplankton, the size of the targeted site and the temporal variation of zooplankton density over a 12-hour period, the overall mean zooplankton density estimate is to be obtained per survey by in-water observers.

## 3. SAFETY

3.1. The ZVI assessments are to be conducted during daylight hours and only when weather conditions are safe for observers to do so (i.e., <7 on the Beaufort scale).

3.2. The buddy system is to be implemented when collecting ZVI estimates.

## 4. EQUIPMENT & DATA RECORDING REQUIREMENTS

4.1 Waterproof notebook.

4.2 Required data: Survey start and finish times. As well as observer name and data grade (researcher- or citizen science-grade).

4.3 Optional data: Estimated abundance of planktivorous animals, environmental parameters, and any relevant contextual information to be captured alongside the ZVI level.

## 5. TRAINING

5.1 All observers undergo training with experienced practitioners to ensure consistency and accuracy in ZVI estimates and to reduce subjectivity across observers. Training consisted of both verbal and written instructions. In addition, conduct in-water training, followed by an in-water assessment (if possible). New observers are required to make three ZVI assessments that match the trainer's assessments before their observations can be considered.

5.2 When in-water training and assessments are not feasible (e.g., citizen scientists), use only verbal and written instructions.


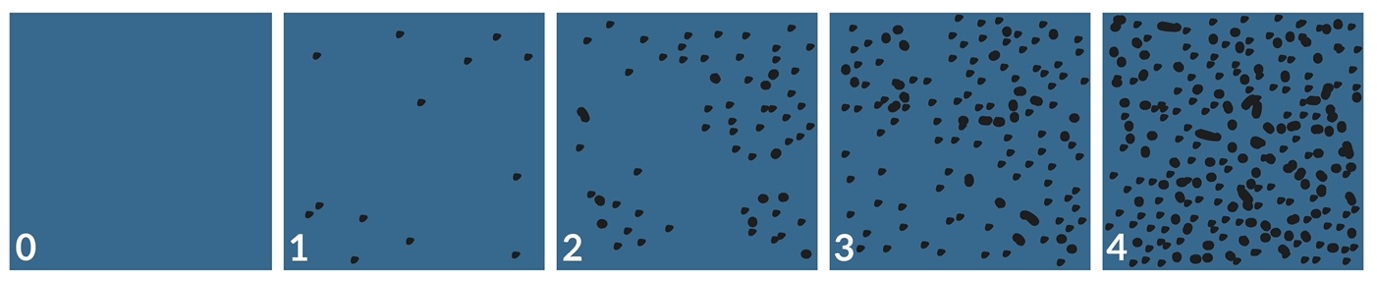


**Figure 1. Standardised density charts used to assess zooplankton visually in the water.** The Zooplankton Visual Index levels on the charts are: (0) apparent absence of zooplankton, (1) thin layer or small patch of zooplankton, (2) multiple layers or patches of zooplankton, (3) water is thick and cloudy, with zooplankton felt on the skin, (4) water is dense and ‘soup-like’.
